# Longitudinal relationship between social and CVD risk factors in older adults with prediabetes: the HRS 2006-2016

**DOI:** 10.18632/aging.206308

**Published:** 2025-08-26

**Authors:** Obinna Ekwunife, Yilin Xu, Raphael Fraser, Jennifer Campbell, Rebekah J. Walker, David Jacobs, Leonard E. Egede

**Affiliations:** 1 Division of Population Health, Department of Medicine, Jacobs School of Medicine and Biomedical Sciences, University at Buffalo, NY 14203, USA; 2 Division of Outcomes and Practice Advancement, Department of Pharmacy Practice, School of Pharmacy, University at Buffalo, Buffalo, NY 14214, USA

**Keywords:** prediabetes, social determinants of health, health equity, cardiovascular health, population health

## Abstract

Background: This study examines how multiple social risk factors influence cardiovascular disease (CVD) risk control over time in older adults with prediabetes using a nationally representative cohort.

Methods: Data from the Health and Retirement Study (HRS) included 5,086 U.S. adults aged 50+ with prediabetes. Five social risk domains (economic stability, environment, education, healthcare, and social context) were examined as independent variables, while CVD risk factors included glycemic control (HbA1c), systolic blood pressure (SBP), and cholesterol ratio (total cholesterol/high-density lipoprotein). Mixed-effects models assessed relationships between social risk factors and CVD outcomes, adjusting for age, gender, race, and marital status.

Results: The sample had an average age of 68.6 years, with 60.2% female, and 70.97% identifying as non-Hispanic Black. Average HbA1c was 5.7, SBP 129.4, and cholesterol ratio 3.85. Limited education was consistently associated with increased CVD risk—HbA1c (β = 0.03, 95% CI: 0.01–0.06, p < 0.001), SBP (β = 4.34, 95% CI: 2.96–5.71, p < 0.001), and cholesterol ratio (β = 0.08, 95% CI: 0.01–0.16, p < 0.05) —in the fully adjusted model. Medication cost-related non-adherence was significantly associated with higher HbA1c levels (β = 0.03, 95% CI: 0.002–0.06, p < 0.05). Difficulty paying bills and lack of health insurance were both significantly associated with higher cholesterol levels (β = 0.03, 95% CI: 0.002–0.06, p < 0.05) and (β = 0.22, 95% CI: 0.15–0.30, p < 0.001), respectively.

Conclusions: Social risk factors, particularly limited education, significantly impact CVD risk in older adults with prediabetes.

## INTRODUCTION

Prediabetes affects approximately 38%, or 97.6 million adults aged 18 or older in the United States [1]. Risk for prediabetes increases with age as an estimated 48% of adults aged 65 and older currently live with prediabetes [1]. Characterized by elevated blood glucose levels that fall short of diabetes criteria (HbA1c ranging from 5.7% to 6.4%), prediabetes is not only a precursor to type 2 diabetes but also increases the risk of cardiovascular disease [2, 3]. Cardiovascular disease (CVD) risk factors include high blood pressure, unhealthy blood cholesterol levels, diabetes mellitus, obesity and smoking [4]. The relationship between prediabetes and CVD risk factors has been well studied, with evidence showing that effective management of these risk factors can significantly mitigate the adverse health outcomes associated with prediabetes and avoid major cardiovascular events [5–8]. However, what is less known is the role that multiple co-occurring social risk factors have in increasing the risk for cardiovascular disease over time among people with prediabetes, particularly for older adults.

Social risk factors, the adverse social conditions that occur within the domains of social determinants of health, are often a function of structural factors and distribution of resources [9]. These include food insecurity, housing instability, limited access to health services and education, as well as social isolation and exposure to violence [9]. Exposure to social risk factors confer significant risk for prediabetes and cardiovascular disease [10, 11]. Specifically, food insecurity has been shown to increase odds of prediabetes by 35% compared to those who are food secure [12]. In addition, exposure to violence and poverty significantly impacts endocrine markers and metabolic health in adolescents, leading to higher rates of prediabetes in adulthood [13]. A review of the evidence for the role of social risk factors in developing CVD found that across social risk factors, social isolation, neighborhood deprivation, discrimination, and violence each increased risk for CVD [14].

While previous studies have explored the individual impact of social risk factors on prediabetes and CVD risk, little has been done to examine the role of multiple social risk factors on CVD risk among individuals living with prediabetes, particularly considering this relationship over time. Moreover, as older adults represent a high-risk population, understanding the relationship between multiple social risk factors on CVD risk among this group is critical. This study examines the association of multiple social risk factors with CVD risk control over time among older adults with prediabetes using a nationally representative cohort. We hypothesize that each social risk domain independently and collectively contributes to poor control of cardiovascular disease (CVD) risk factors over time, even after adjusting for relevant covariates.

## MATERIALS AND METHODS

### Dataset

The Health and Retirement Study (HRS, 2024) is a longitudinal panel study initiated in 1992 by the University of Michigan’s Institute for Social Research. It surveys a representative sample of over 20,000 Americans aged 50 and older every two years, focusing on the multifaceted aspects of aging [15]. The HRS collects comprehensive data on health status, chronic conditions (such as diabetes and prediabetes), healthcare utilization, employment, and wealth, as well as physical measures and genetic markers. Prediabetes information is captured through self-reported measures and biomarkers, including blood glucose levels and hemoglobin A1c.

### Study population

The study population was drawn from HRS core interviews conducted from 2006 to 2016. The study included individuals aged 50 or older who also participated in the HRS biomarker project. Participants were eligible if they had no prior diagnosis of diabetes, and their glycemic control (HbA1c) levels were greater or equal to 5.7% and lower than 6.5%. Interviews were conducted in two groups with three collection points for each: group 1 was assessed in 2006, 2010, and 2014, and group 2 was assessed in 2008, 2012, and 2016. Data was compiled from both groups to create a single sample of adults with multiple time points of data collection. Among 11,573 older adults who participated in the Health and Retirement Study (HRS) and the HRS biomarker project, 5,086 had prediabetes and were included in all analyses.

### Variables

### Primary independent variables

The study examined five social risk domains outlined by the Kaiser Family Foundation social determinants framework as primary independent variables based on variables available in the dataset: economic stability, neighborhood or built environment, education access, health care access, and social or community context [16, 17].

- Economic Stability: Assessed using four questions: difficulty paying bills, skipping medications due to cost, being in the lowest quartile for income or assets, and employment adversity.
  - Difficulty paying bills was determined from the question: “How difficult is it for (you/your family) to meet monthly payments on (you/your family’s) bills?”. If the response was “not at all” or “not very difficult”, it was coded as no difficulty paying bills; otherwise, it was coded as having difficulty paying bills.
  - Medication cost-related nonadherence was assessed using the question: “Have you ever taken less medication because of cost?” If the answer was “yes,” it was coded as yes for medication cost-related nonadherence; otherwise, it was coded as no medication cost-related nonadherence.
  - Lowest quartile for income or assets was determined by summing responses to total assets and average household income. This sum was divided into four quartiles, and individuals in the lowest quartile were coded as “yes”; otherwise, they were coded as “no”.
  - Employment adversity was identified based on the question: “What is your current job status?” Respondents who answered “unemployed and looking for work,” “temporarily laid off,” or “disabled” were coded as “yes” for employment adversity; otherwise, they were coded as “no”.
- Neighborhood or Built Environment: Evaluated using four variables: food insecurity, neighborhood physical disorder, lack of neighborhood social cohesion, and adverse social support.
  - Food insecurity was assessed using two questions: “Have you always had enough money to buy the food you need?” and “Did you ever eat less than you felt you should because there wasn’t enough money to buy food?” Respondents who answered “no” to the first question or “yes” to the second question were coded as experiencing food insecurity.
  - Neighborhood physical disorder was measured using four survey items assessing vandalism and graffiti, safety when walking alone at night, cleanliness, and the presence of vacant properties. Each item was rated on a 1 to 7 scale, where lower values indicated better neighborhood conditions and higher values indicated worse conditions. A neighborhood disorder score was calculated as the meaning of these responses. If the score was 4 or higher, the respondent was classified as experiencing neighborhood physical disorder; otherwise, they were classified as not experiencing neighborhood physical disorder.
  - Neighborhood social cohesion was assessed using four survey items evaluating feelings of belonging, trust in neighbors, friendliness, and neighborhood support. Responses were recorded on a 1 to 7 scale, where lower values indicated stronger cohesion. To standardize interpretation, the social cohesion score was reverse-coded by subtracting the mean response from 8, so that higher values represented stronger cohesion. The score was calculated only for respondents who answered at least two of the four items. Those with scores between 0 and 4 were classified as experiencing low social cohesion, while those with scores above 4 were classified as having strong social cohesion.
  - Adverse social support was measured based on both positive and negative support from spouses/partners, children, other family members, and friends. Positive support was assessed through questions about feeling understood, reliability, and communication openness, while negative support included excessive demands, criticism, disappointment, and annoyance. A positive support score was computed as the average of three relevant responses, and a negative support score was computed using four relevant responses. Both scores were reverse-coded to ensure higher values indicated stronger positive support and weaker negative support. A final social support score was calculated by combining positive and negative support measures. Respondents with a score below 3 were classified as having adverse social support, while those with a score of 3 or higher were classified as having sufficient social support.
- Education Access: Determined based on respondents’ highest level of education. Those without a high school diploma were categorized as having low education access.
- Health Care Access: Assessed based on whether respondents lacked health insurance. This was determined using four questions about coverage through employment insurance, government insurance, other private insurance, or long-term care insurance. If respondents answered “yes” to any of these coverage options, they were classified as having health care access; otherwise, they were classified as lacking health care access.
- Social or Community Context: Assessed using two indicators: depression and perceived everyday discrimination.
  - Depression was identified using the CES-D score, where respondents with a score of 4 or higher were classified as experiencing depression, while those with scores between 0 and 3 were classified as not experiencing depression [17].
  - Everyday discrimination was measured using six survey items that captured experiences of being treated with less respect, receiving poorer service, being perceived as unintelligent, causing fear in others, being harassed or threatened, and receiving worse treatment in healthcare settings. Each item was rated on a 1 to 6 scale, where lower values indicated more frequent discrimination and higher values indicated rare or no experiences of discrimination. The discrimination score was calculated only for respondents who answered at least three of the six items. To standardize interpretation, the score was reverse-coded by subtracting the mean response from 7, ensuring that higher values indicated more frequent discrimination. Respondents with scores of 2 or higher were classified as experiencing frequent discrimination, while those with scores below 2 were classified as not experiencing frequent discrimination.

Each social risk domain was coded as a binary variable, with responses categorized as either “yes” (exposed to risk) or “no” (not exposed to risk). For domains where more than one indicator was incorporated, exposure to any of the indicators was considered exposed to risk.

### Covariates

We adjusted for several covariates in the analysis that are well-established confounders in the relationship between social risk factors and CVD risk. The covariates included both demographic factors and comorbidities. The demographic factors included age groups (50–59, 60–74, and 75+ years), sex (male or female), race/ethnicity (Non-Hispanic Black, Non-Hispanic White, Other), and marital status (married or not). These factors are known to influence both social conditions and cardiovascular health outcomes. For example, age and gender are independently associated with CVD risk, while race and marital status can shape social exposure and access to care. The comorbidities included were high blood pressure, cancer (excluding skin cancer), lung disease, heart disease, stroke, emotional or psychiatric problems, and arthritis, to assess their potential confounding impact. Heart disease was identified through four questions about doctor diagnoses, heart attacks, chest pain, and congestive heart failure. Each comorbidity was treated as a separate variable in the models. These variables were considered based on previous literature indicating their relevance to both social risk and CVD progression.

### Primary outcomes

Three continuous primary outcomes were assessed: Hemoglobin A1c (HbA1c), systolic blood pressure (SBP), and cholesterol ratio [17]. HbA1c was measured using NHANES-equivalent data from the HRS Biomarker file. Systolic blood pressure was represented by a value calculated from three measurements for each individual. When only one value was available, it was used; when multiple values were present, the average was calculated. The cholesterol ratio was calculated by dividing total cholesterol by high-density lipoprotein (HDL) cholesterol, using NHANES-equivalent data from the Biomarker file.

### Statistical analysis

Descriptive statistics for participant characteristics were summarized as means and standard deviations for continuous variables, and as counts and percentages for categorical variables, for individuals with prediabetes [15]. We employed mixed-effects linear models to estimate the effects of social risk factors on glycemic control, systolic blood pressure, and the cholesterol ratio (total cholesterol to high-density lipoprotein) [18]. In the mixed-effects models, participant-specific random effects accounted for repeated measures across time, with the unique participant ID serving as the random intercept. Time was represented by the survey wave to capture longitudinal trends. While more complex models incorporating random slopes or time-varying interactions may capture individual-specific trajectories or evolving social risk effects over time, our primary objective was to estimate population-averaged associations between social risk domains and outcomes across the study period. Thus, we opted for a parsimonious modeling strategy focused on main effects, which balances interpretability with power and model stability. Potential interactions between social risk factors were not included in the current analysis due to limited power to detect higher-order interactions and concerns regarding multicollinearity among domains. Each outcome was treated as a separate set of models with seven models running for each. First, five separate models were run with the five social risk domains individually added. Second, a model was run with all five social risk domains added together. Finally, a model was run with all five social risk domains and covariates. To account for the complex survey design and ensure generalizability to the U.S. population, all analyses incorporated sampling weights provided by the Health and Retirement Study [15]. Model performance was evaluated using appropriate goodness-of-fit measures, with statistical significance set at a p-value of less than 0.05. All statistical analyses were performed using Stata software, version 16.0 [19].

## RESULTS

Table 1 summarizes the sample characteristics. The average age of participants was 68.6 years (SD = 10.3). Among them, 2,026 (39.8%) were male, and 3,060 (60.2%) were female. The majority of the cohort, 70.97%, identified as non-Hispanic Black. A total of 3,500 (68.8%) participants were married or living with a partner. The average hemoglobin A1c was 5.78 (SD = 0.35), the mean systolic blood pressure was 129.4 (SD = 19.5), and the average cholesterol ratio was 3.85 (SD = 1.15).

**Table 1:** Sample characteristics of older adults with prediabetes in the health and retirement study, January 2006 to December 2016 (n=5,086).

	**No. (%)**
**Age**, year, mean (SD)	68.63 (10.27)
50-59	1685 (33.13)
60-74	2806 (55.17)
≥75	1851 (36.39)
**Sex**	
Male	2026 (39.83)
Female	3060 (60.17)
**Race and ethnicity**	
Non-Hispanic Black	3609 (70.97)
Non-Hispanic White	814 (16.01)
Other	662 (13.02)
**Married or living with a partner**	3500 (68.82)
**Comorbidities**	
High blood pressure	3071 (60.41)
Cancer	892 (17.55)
Lung disease	631 (12.41)
Heart disease	1370 (26.95)
Stroke	344 (6.76)
Emotional or psychiatric problems	917 (18.03)
Arthritis	3238 (63.69)
**Economic stability**	
Medication cost-related nonadherence	690 (13.57)
Difficulty paying bills	1960 (38.54)
Lowest quartile income or assets	1206 (23.71)
Employment adversity	687 (13.51)
**Neighborhood or built environment**	
Food insecurity	320 (6.29)
Neighborhood physical disorder	1284 (25.25)
Lack of neighborhood social cohesion	1249 (24.56)
Adverse social support	1577 (31.01)
**Education access**	
Limited education	814 (16.00)
**Health care access**	
Has Health insurance	4599 (90.42)
**Social or community context**	
Depression	882 (17.34)
Perceived everyday discrimination	1583 (31.12)
**Outcomes**	
**Blood hemoglobin (HbA1c)**, Mean (SD)	5.78 (0.35)
**Systolic blood pressure (SBP)**, Mean (SD)	129.41 (19.46)
**Cholesterol ratio, Mean** (SD)	3.85 (1.15)

**Figure 1:**
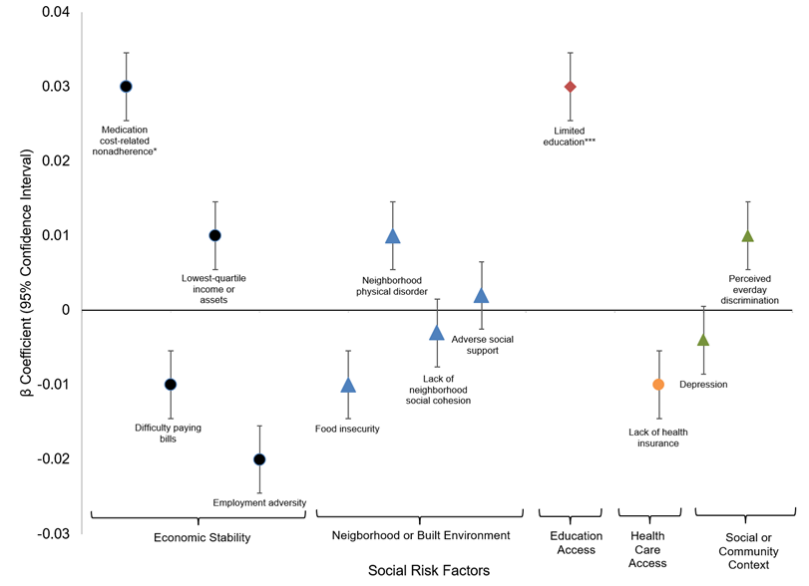
**Fully adjusted mixed effects linear regression for relationship between social risk factors and glycemic control.** * p-value <0.05; **p-value <0.01; *** p-value <0.001.

**Figure 2:**
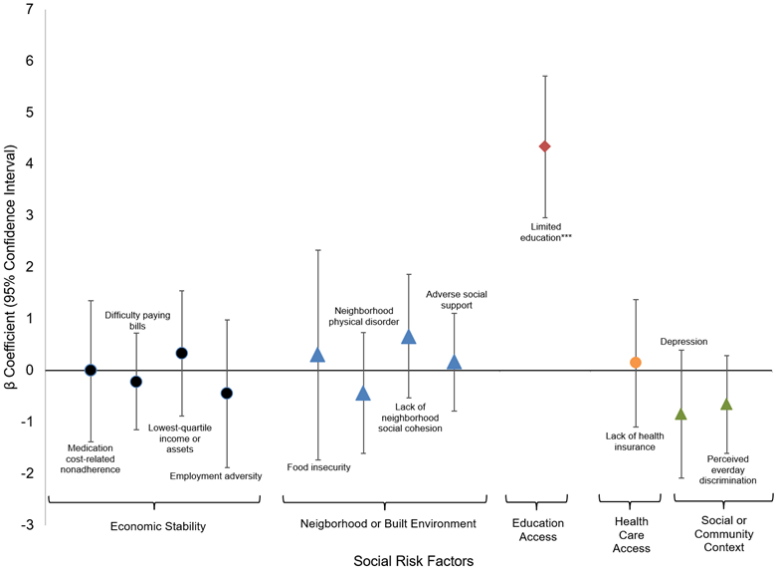
**Fully adjusted mixed effects linear regression for relationship between social risk factors and systolic blood pressure.** * p-value <0.05; **p-value <0.01; *** p-value <0.001.

**Figure 3:**
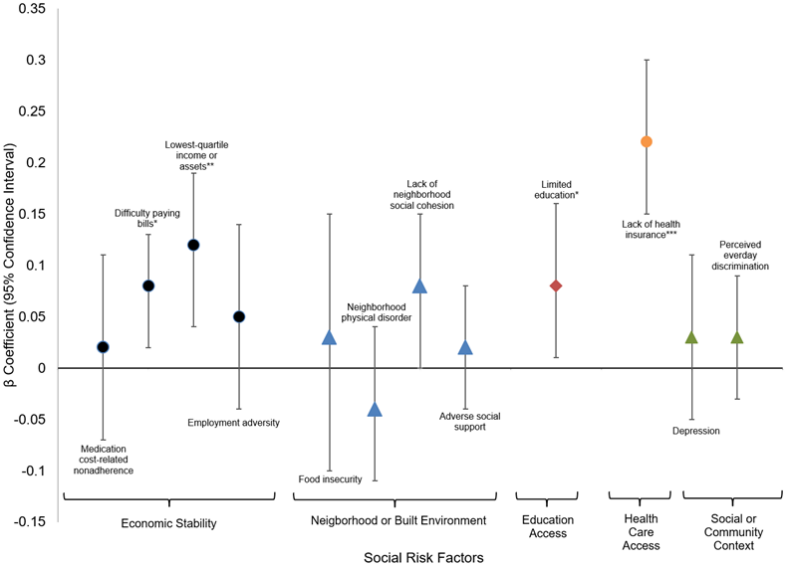
**Fully adjusted mixed effects linear regression for relationship between social risk factors and cholesterol ratio.** * p-value <0.05; **p-value <0.01; *** p-value <0.001.

Figures 1–3 present the beta coefficients and 95% confidence intervals from fully adjusted mixed-effects linear regression models examining the association between social risk domains and three cardiometabolic outcomes: glycemic control (Figure 1), systolic blood pressure (Figure 2), and total cholesterol to HD ratio (Figure 3). Furthermore, the appendix (Supplementary Tables 1–3) show the results of the three sets of mixed-effects linear regression models: the unadjusted model for each social risk domains, the adjusted model including all five social risk domains, and the fully adjusted model includes all five social risk domains and additional covariates. In Figure 1, the results highlight significant associations between social risk domains, demographic characteristics, clinical covariates, and HbA1c. Within the Economic Stability domain, medication nonadherence due to cost was associated with a small but significant increase in HbA1c across all models (β = 0.03, 95% CI: 0.002–0.06, p < 0.05 in the fully adjusted model). Within the Education domain, low education was significantly associated with higher HbA1c, even after adjusting for covariates in the fully adjusted model (β = 0.03, 95% CI: 0.01–0.06, p < 0.001).

Figure 2 shows the associations between social risk domains, demographic characteristics, clinical covariates, and systolic blood pressure (SBP). Low education was significantly associated with higher SBP across all models: unadjusted model (β = 4.85, 95% CI: 3.50–6.20, p < 0.001), adjusted model (β = 5.03, 95% CI: 3.64–6.42, p < 0.001), and fully adjusted model (β = 4.34, 95% CI: 2.96–5.71, p < 0.001).

Figure 3 presents the associations between social risk domains, demographic characteristics, clinical covariates, and cholesterol ratio. Within the Economic Stability domain, difficulty paying bills was significantly associated with a higher cholesterol ratio in the fully adjusted model (β = 0.08, 95% CI: 0.02–0.13, p < 0.05), and lowest-quartile income or assets also showed a significant positive association (β = 0.12, 95% CI: 0.04–0.19, p < 0.01). Low education was significantly associated with higher cholesterol ratios, with an effect of (β = 0.08, 95% CI: 0.01–0.16, p < 0.05) in the fully adjusted model. Lack of health insurance was significantly associated with a higher cholesterol ratio in all models, with the strongest effect in the fully adjusted model (β = 0.22, 95% CI: 0.15–0.30, p < 0.001).

## DISCUSSION

This study is the first to examine the long-term impact of multiple social risk factors on cardiovascular health risk factors—HbA1c, systolic blood pressure (SBP), and cholesterol ratios—among older adults with prediabetes in the United States using nationally representative longitudinal data from the Health and Retirement Study (HRS), addressing a key gap in the literature on the interaction of multiple determinants. The analysis revealed complex interactions between social risk factors, cardiovascular health outcomes, demographic characteristics, and clinical conditions over time. Limited education was the only social risk factor consistently associated with increased risk across all three outcomes—HbA1c, systolic blood pressure (SBP), and cholesterol ratios—in the fully adjusted model (i.e., after adjustment for demographic and clinical covariates). Within the domain of economic stability, medication cost related non-adherence was significantly associated with HbA1c and difficulty paying bills was significantly associated with higher cholesterol ratios in the fully adjusted model. Healthcare access, determined through lack of health insurance, was also associated with higher cholesterol ratios in fully adjusted model. Consistent with our findings, studies have linked individual social risk factors to cardiovascular health in people with or without prediabetes. For example, well-maintained neighborhoods are associated with physical activity, improved insulin sensitivity and lower HbA1c [20], while racial discrimination in healthcare is associated with higher HbA1c in adults with diabetes [21]. This study adds to the literature by providing insight on which social risks are independently associated with clinical outcomes in adults with prediabetes when accounting for other social risks, demographic, and clinical factors.

Limited education was the only social risk factor consistently associated with worse outcomes (HbA1c, systolic blood pressure, and cholesterol ratios) after full adjustment for demographic and clinical factors. Other studies have identified associations with higher HbA1c levels [22, 23], higher systolic blood pressure over 30 years, with a potentially stronger effect in females than males [24], and higher lipid profiles, particularly in women [25]. Our findings add to this literature by showing that, even after accounting for other social risks, limited education is an independent risk for poor outcomes in adults with prediabetes. More research is needed on how to mitigate this risk through structural interventions targeting improved educational opportunities. In addition, research should focus on understanding the mechanism through which limited education has an influence on prediabetes given the possible pathways of limited income due to educational level, environmental exposures due to job type, and behavioral activities based on limited understanding of health.

Economic instability, particularly medication nonadherence due to cost and low income or assets, was significantly associated with higher HbA1c levels and high cholesterol, even after adjusting for other social risk domains and covariates. This highlights the importance of financial stability in managing prediabetes. These findings align with previous research showing that financial stress contributes to medication nonadherence in individuals with chronic conditions, including diabetes [26, 27]. Additionally, similar associations between financial strain and elevated cholesterol levels have been reported in other studies [28]. Addressing economic barriers may improve medication adherence and, consequently, glycemic and cholesterol control in individuals with prediabetes. Expanding affordable healthcare, enhancing food security, and leveraging Medicaid waivers are essential strategies to mitigate economic barriers [9, 29, 30].

Finally, lack of health insurance was associated with higher cholesterol levels in individuals with prediabetes after adjusting for other social risk factors and relevant demographic and clinical covariates. This could be due to barriers in accessing routine medical care and preventive therapies. Uninsured individuals face a greater risk of undiagnosed and untreated dyslipidemia, while those with insurance are more likely to receive screenings and manage risk factors effectively [31, 32]. Improving health insurance coverage may enhance access to preventive care, enabling earlier detection and management of elevated cholesterol in individuals with prediabetes. Future interventions studies should focus on eliminating insurance-related barriers in people with prediabetes and measuring their impact on cardiovascular health outcomes. Examples of interventions to eliminate insurance-related barriers include capping out-of-pocket costs for essential services, regulating prescription drug pricing and promoting generics, providing community-based health insurance navigators, increasing Affordable Care Act subsidies, and using Medicaid waivers for innovative coverage solutions.

The findings of this study have several important clinical implications. First, there remains a need for interventions targeting key social risk factors to improve cardiovascular outcomes in adults with prediabetes. Strategies such as community-based health education initiatives and economic support programs and policies addressing systemic inequities could play an important role in reducing health disparities among older adults. Based on these findings, tailored diabetes prevention interventions for high-risk groups, such as those with limited education or economic resources, are particularly important for bridging health equity gaps. This approach aligns with the National DPP’s commitment to advancing health equity by expanding access to lifestyle change program for priority populations [33]. One strategy for tailoring diabetes prevention intervention is to address economic barriers, such as providing subsidies for healthy food and promoting physical activity [34, 35]. Additionally, simplifying educational materials, leveraging technology for flexible program access, and integrating peer-led support systems can enhance engagement and long-term adherence [36, 37]. Secondly, expanding efforts to integrate social and medical care into healthcare policy and practice—such as screening for social needs, implementing Medicaid waivers to provide non-medical services like transportation and housing, and offering training programs that emphasize the importance of social determinants while equipping providers to address them—could significantly advance health equity and improve population health [9, 29, 30]. Community health workers (CHWs) or trained navigators could help individuals access resources and support, addressing social needs that affect health outcomes and therefore should be used more widely [38]. Currently, prediabetes is not considered a priority diagnosis for Medicaid waiver programs that provide non-medical services. These findings suggest the need to include prediabetes in future iterations of waiver programs or those developed based on Medicaid waiver demonstration projects.

Despite incorporating multiple social risks and using a nationally representative dataset collected over time, this study has some limitations to note. First, generalizability of these findings is limited to older adults with prediabetes in the United States and may not extend to other regions (such as developing countries) or to younger populations with prediabetes. Additional work is needed to identify if similar patterns hold in other age groups of adults with prediabetes. Second, while this study identifies significant associations given consideration of other social risks, it does not investigate possible mechanisms of this effect. Future research should explore the mechanisms linking social risk factors with health outcomes to guide interventional research. Third, our model did not include certain lifestyle and environmental variables, such as dietary structure, exercise frequency, and air pollution exposure, due to limitations in the available data. The absence of these factors may result in residual confounding, potentially leading to an overestimation of the independent effects of social risk factors. Future studies with more detailed data on these exposures are needed to validate and refine these findings. Fourth, this study did not include measures of locomotory restriction, which may influence both social risk exposure and cardiovascular outcomes among older adults. Also, social risk factors incorporated in this analysis were limited to the variables collected in the HRS dataset. Longitudinal studies with additional social risk factors and more granular data on social determinants of health domains could provide deeper insights into modifying health disparities. Additional limitations include potential survival bias, as the sample only includes individuals who survived to participate and had follow-up data, possibly underestimating the impact of social risk among those with early mortality. Self-reported social risk variables may be subject to recall bias, leading to misclassification and attenuation of observed associations.

## CONCLUSIONS

This study highlights the significant influence of social risk factors on cardiovascular health outcomes in older adults with prediabetes. After accounting for five domains of social risk, education and economic stability domains were the most consistently associated with glycemic control, blood pressure, and cholesterol levels. These findings underscore the need for targeted interventions to address social risk factors in improving cardiovascular outcomes in adults with prediabetes. Expanding tailored diabetes prevention programs and integrating social care into healthcare policies for high-risk groups, particularly for those with limited education or economic resources, have promise to enhance engagement and promote health equity. Additionally, prioritizing prediabetes in future healthcare initiatives, including Medicaid waivers, could further improve population health outcomes.

## Supplementary Material

Supplementary Tables
