# Supplementary material for: Longitudinal relationship between social and CVD risk factors in older adults with prediabetes: the HRS 2006-2016
Source: Aging (Albany NY). 2025 Aug 26;17(8):2152–66. doi: 10.18632/aging.206308 (PMC12422790; doi:10.18632/aging.206308)
Supplement: Supplementary Tables [file aging-17-8-206308-s001.pdf]

SUPPLEMENTARY TABLES

Supplementary Table 1. Adjusted mixed effects linear regression for relationship between social risk domains and glycemic control.

	Model 1 [2] B coefficient (95% confidence interval)	Model 2 [3] B coefficient (95% confidence interval)	Model 3 [4] B coefficient (95% confidence interval)
**Economic stability**			
Medication cost-related nonadherence	**0.03 (0.005, 0.06) ***	**0.03 (0.002, 0.06) ***	**0.03 (0.002, 0.06) ***
Difficulty paying bills	-0.001 (-0.02, 0.02)	-0.004 (-0.02, 0.01)	-0.01 (-0.02, 0.01)
Lowest-quartile income or assets	**0.05 (0.03, 0.07) *****	**0.03 (0.01, 0.05) *****	0.01 (-0.01, 0.03)
Employment adversity	-0.02 (-0.04, 0.01)	-0.02 (-0.04, 0.01)	-0.02 (-0.05, 0.003)
**Neighborhood or built environment**			
Food insecurity	0.02 (-0.02, 0.06)	0.00 (-0.04, 0.04)	-0.01 (-0.05, 0.03)
Neighborhood physical disorder	**0.03 (0.01, 0.05) ****	0.02 (-0.001, 0.04)	0.01 (-0.01, 0.03)
Lack of neighborhood social cohesion	0.01 (-0.01, 0.04)	0.01 (-0.02, 0.03)	-0.003 (-0.03, 0.02)
Adverse social support	0.003 (-0.01, 0.02)	-0.001 (-0.02, 0.02)	0.002 (-0.02, 0.02)
**Education access**			
Limited education	**0.07 (0.05, 0.09) *****	**0.06 (0.04, 0.08) *****	**0.03 (0.01, 0.06) *****
**Health care access**			
Lack of health insurance	-0.02 (-0.04, 0.00)	**-0.03 (-0.05, -0.01) ****	-0.01 (-0.04, 0.01)
**Social or community context**			
Depression	0.02 (-0.01, 0.04)	0.002 (-0.02, 0.02)	-0.004 (-0.03, 0.02)
Perceived everyday discrimination	0.01 (-0.01, 0.03)	0.01 (-0.01, 0.03)	0.01 (-0.01, 0.02)
**Covariates**			
High blood pressure	-	-	**0.03 (0.01, 0.04) *****
Cancer	-	-	**0.03 (0.01, 0.05) ****
Lung disease	-	-	**0.04 (0.01, 0.06) ****
Heart disease	-	-	**0.02 (0.003, 0.04) ***
Stroke	-	-	0.03 (-0.001, 0.06)
Emotional or psychiatric problems	-	-	-0.01 (-0.03, 0.01)
Arthritis	-	-	-0.01 (-0.02, 0.01)
**Age**, year (Ref: 50-59)			
60-74	-	-	-0.01 (-0.03, 0.01)
≥75	-	-	**0.04 (0.02, 0.06) *****
**Race and ethnicity** (Ref: Non-Hispanic White)			
Non-Hispanic Black	-	-	**0.12 (0.10, 0.14) *****
Other	-	-	**0.08 (0.05, 0.10) *****
**Married or living with a partner** (Ref: single)	-	-	-0.004 (-0.02, 0.01)
**Sex** (Ref: Male)			**-0.02 (-0.03, -0.002) ***

[1]*p-value <0.05; **p-value <0.01; *** p-value <0.001.

[2]This model includes five separate individual models, each containing only one of the social risk factors.

[3]This model includes all five social risk factors together.

[4]This model includes all five social risk factors along with covariates.

Supplementary Table 2. Adjusted mixed effects linear regression for relationship between social risk domains and systolic blood pressure.

	Model 1[2] B coefficient (95% confidence interval)	Model 2[3] B coefficient (95% confidence interval)	Model 3[4] B coefficient (95% confidence interval)
**Economic stability**			
Medication cost-related nonadherence	-0.63 (-1.99, 0.73)	-0.48 (-1.86, 0.89)	-0.01 (-1.38, 1.35)
Difficulty paying bills	-0.86 (-1.78, 0.07)	-0.69 (-1.63, 0.26)	-0.22 (-1.15, 0.72)
Lowest-quartile income or assets	**1.45 (0.31, 2.59) ***	0.75 (-0.43, 1.94)	0.33 (-0.88, 1.55)
Employment adversity	**-1.59 (-2.99, -0.17) ***	-1.23 (-2.66, 0.20)	-0.45 (-1.89, 0.98)
**Neighborhood or built environment**			
Food insecurity	-0.61 (-2.59, 1.36)	-0.09 (-2.16, 1.97)	0.30 (-1.74, 2.33)
Neighborhood physical disorder	0.16 (-1.02, 1.34)	-0.12 (-1.31, 1.06)	-0.44 (-1.61, 0.74)
Lack of neighborhood social cohesion	0.55 (-0.66, 1.75)	0.60 (-0.61, 1.81)	0.66 (-0.53, 1.86)
Adverse social support	-0.76 (-1.70, 0.18)	-0.39 (-1.36, 0.57)	0.16 (-0.79, 1.11)
**Education access**			
Limited education	**4.85 (3.50, 6.20) *****	**5.03 (3.64, 6.42) *****	**4.34 (2.96, 5.71) *****
**Health care access**			
Lack of health insurance	**-2.17 (-3.35, -0.98) *****	**-2.01 (-3.21, -0.82) ****	0.14 (-1.10, 1.37)
**Social or community context**			
Depression	-1.01 (-2.21, 0.18)	-1.12 (-2.34, 0.11)	-0.85 (-2.09, 0.39)
Perceived everyday discrimination	**-1.21 (-2.14, -0.28) ****	**-0.98 (-1.94, -0.03) ***	-0.66 (-1.61, 0.29)
**Covariates**			
High blood pressure	-	-	**4.87 (3.98, 5.77) *****
Cancer	-	-	**-1.30 (-2.50, -0.10) ***
Lung disease	-	-	**-1.66 (-3.07, -0.25) ***
Heart disease	-	-	**-3.47 (-4.50, -2.43) *****
Stroke	-	-	0.12 (-1.74, 1.98)
Emotional or psychiatric problems	-	-	-0.71 (-1.95, 0.54)
Arthritis	-	-	-0.15 (-1.07, 0.77)
**Age**, year (Ref: 50-59)			
60-74	-	-	**3.18 (2.14, 4.22) *****
≥75	-	-	**6.85 (5.55, 8.16) *****
**Race and ethnicity** (Ref: Non-Hispanic White)			
Non-Hispanic Black	-	-	**3.43 (2.00, 4.86) *****
Other	-	-	**-2.27 (-3.80, -0.74) ****
**Married or living with a partner** (Ref: single)	-	-	**-1.13 (-2.11, -0.15) ***
**Sex** (Ref: Male)			**-4.29 (-5.29, -3.29) *****

[1]*p-value <0.05; **p-value <0.01; *** p-value <0.001.
[2]This model includes five separate individual models, each containing only one of the social risk factors.
[3]This model includes all five social risk factors together.
[4]This model includes all five social risk factors along with covariates.

Supplementary Table 3. Adjusted mixed effects linear regression for relationship between social risk domains and cholesterol ratio.

	**Model 1 [2]** B coefficient (95% confidence interval)	**Model 2 [3]** B coefficient (95% confidence interval)	**Model 3 [4]** B coefficient (95% confidence interval)
**Economic stability**			
Medication cost-related nonadherence	-0.001 (-0.09, 0.08)	-0.02 (-0.11, 0.06)	0.02 (-0.07, 0.11)
Difficulty paying bills	**0.10 (0.05, 0.16)** ***	**0.07 (0.01, 0.13)** *	**0.08 (0.02, 0.13)** *
Lowest-quartile income or assets	**0.09 (0.02, 0.16)** **	0.06 (-0.01, 0.13)	**0.12 (0.04, 0.19)** **
Employment adversity	**0.11 (0.02, 0.19)** *	0.07 (-0.02, 0.16)	0.05 (-0.04, 0.14)
**Neighborhood or built environment**			
Food insecurity	0.11 (-0.01, 0.23)	0.01 (-0.11, 0.14)	0.03 (-0.10, 0.15)
Neighborhood physical disorder	-0.03 (-0.10, 0.05)	-0.05 (-0.12, 0.02)	-0.04 (-0.11, 0.04)
Lack of neighborhood social cohesion	**0.10 (0.03, 0.18)** **	0.07 (-0.01, 0.14)	0.08 (-0.00, 0.15)
Adverse social support	**0.08 (0.02, 0.13)** **	0.04 (-0.02, 0.10)	0.02 (-0.04, 0.08)
**Education access**			
Limited education	**0.13 (0.05, 0.20)** **	**0.08 (0.01, 0.16)** *	**0.08 (0.01, 0.16)** *
**Health care access**			
Lack of health insurance	**0.29 (0.22, 0.37)** ***	**0.26 (0.19, 0.33)** ***	**0.22 (0.15, 0.30)** ***
**Social or community context**			
Depression	0.03 (-0.04, 0.11)	-0.03 (-0.10, 0.05)	0.03 (-0.05, 0.11)
Perceived everyday discrimination	**0.10 (0.04, 0.16)** **	**0.06 (-0.001, 0.12)** *	0.03 (-0.03, 0.09)
**Covariates**			
High blood pressure	-	-	-0.01 (-0.06, 0.04)
Cancer	-	-	-0.05 (-0.12, 0.02)
Lung disease	-	-	-0.00 (-0.08, 0.08)
Heart disease	-	-	**-0.15 (-0.21, -0.09)** ***
Stroke	-	-	-0.06 (-0.17, 0.05)
Emotional or psychiatric problems	-	-	-0.005 (-0.08, 0.07)
Arthritis	-	-	0.01 (-0.04, 0.07)
**Age**, year (Ref: 50-59)			
60-74	-	-	0.03 (-0.03, 0.09)
≥75	-	-	**-0.09 (-0.17, -0.01)** *
**Race and ethnicity** (Ref:Non-Hispanic White)			
Non-Hispanic Black	-	-	**-0.12 (-0.20, -0.04)** **
Other	-	-	0.03 (-0.05, 0.12)
**Married or living with a partner** (Ref: single)	-	-	0.02 (-0.04, 0.08)
**Sex** (Ref: Male)			**-0.37 (-0.43, -0.32)** ***

[1]*p-value <0.05; **p-value <0.01; *** p-value <0.001.
[2]This model includes five separate individual models, each containing only one of the social risk factors.
[3]This model includes all five social risk factors together.
[4]This model includes all five social risk factors along with covariates.
